# Cerebellar Mutism Treated Successfully With Zolpidem in a Patient With Learning Difficulties

**DOI:** 10.7759/cureus.16616

**Published:** 2021-07-25

**Authors:** Rana Moshref, Abeer Mirdad

**Affiliations:** 1 Surgery, King Abdulaziz University, Jeddah, SAU; 2 Pediatrics, East Jeddah Hospital, Jeddah, SAU

**Keywords:** cerebellar mutism, zolpidem, astrocytoma, posterior fossa tumor, pediatrics

## Abstract

Posterior fossa tumors constitute the most common brain tumor in pediatrics with 25% development postresection. Cerebellar mutism can manifest as neurobehavioral abnormalities that can occur within days to months after surgery but usually peak on the third postoperative day. It can be caused by discontinuation of dento-thalamo-cortical pathway in the vermian lesion, due to edema, tumors, and hypoperfusion. We report a seven-year-old patient with posterior fossa lesion (pilocytic astrocytoma in histopathology) and learning difficulties with symptoms of urinary retention, pseudobulbar palsy, and motor incoordination that were treated successfully with zolpidem 2.5 mg with regain of function by the third month.

## Introduction

Posterior fossa tumors are the most frequently encountered brain tumors in pediatrics [1]. One complication of resection of these tumors is cerebellar mutism, which develops in 25% of these patients, with the highest occurrence for medulloblastoma, followed by astrocytoma and ependymoma [1]. Cerebellar mutism can manifest as neurobehavioral abnormalities, personality changes, emotional lability, poor oral intake, dysarthria, ataxia, and urinary incontinence [2,3] that can occur within days to months after surgery but usually peak on the third postoperative day [3]. The tumors, edema, hypoperfusion, and axonal injury in the vermis are postulated to cause disruption of the dento-thalamo-cortical pathway, especially in cases with high-grade tumors and in young patients with language impairment [4]. Some therapies such as corticosteroid, fluvoxamine, zolpidem, aripiprazole, and lorazepam have been tried, although only in case reports, favorable outcomes are reported for zolpidem and fluvoxamine [5,6].

Zolpidem is used as a sedative, but it has been used to treat psychiatric disorders. As per our knowledge, there are only two cases (children of normal development) that improved with zolpidem in terms of language and movement with regain of baseline function by one month [7]. Here, we present a unique case of zolpidem treatment of cerebellar mutism in a patient who is mentally challenged.

## Case presentation

Medical history

**Figure 1:**
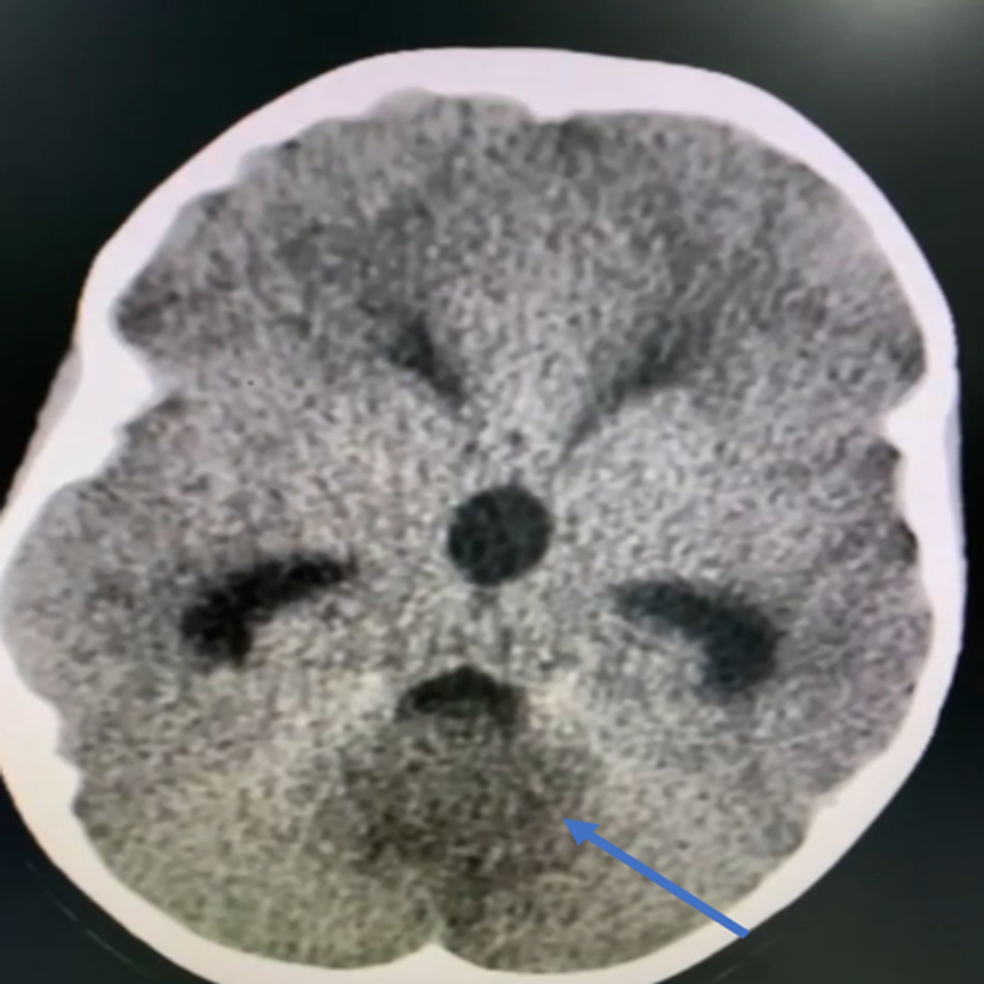
Preoperative image - CT brain axial cut shows obstructive hydrocephalus showing posterior fossa lesion

A seven-year-old girl who was medically and surgically free presented to our ER complaining of headache and vomiting for one month. CT images done in another hospital showed a posterior fossa lesion (Figure 1). The headache was frontal and bitemporal upon waking from sleep, according to the mother, and projectile vomiting of food occurred several times a day, sometimes upon waking from sleep. These symptoms were associated with significant weight loss, loss of appetite, and night sweats for one month, along with diplopia, blurred vision, gait ataxia, and excessive yawning. The child had no history of seizures, no trauma, and no reported weakness or sensory loss. The family and social history showed a positive family history of leukemia and uterine cancers, consanguineous parents, and five siblings (three girls and two boys) with hearing impairment living in the same housing. The child had learning problems in school, according to the mother.

Clinical findings

**Figure 2:**
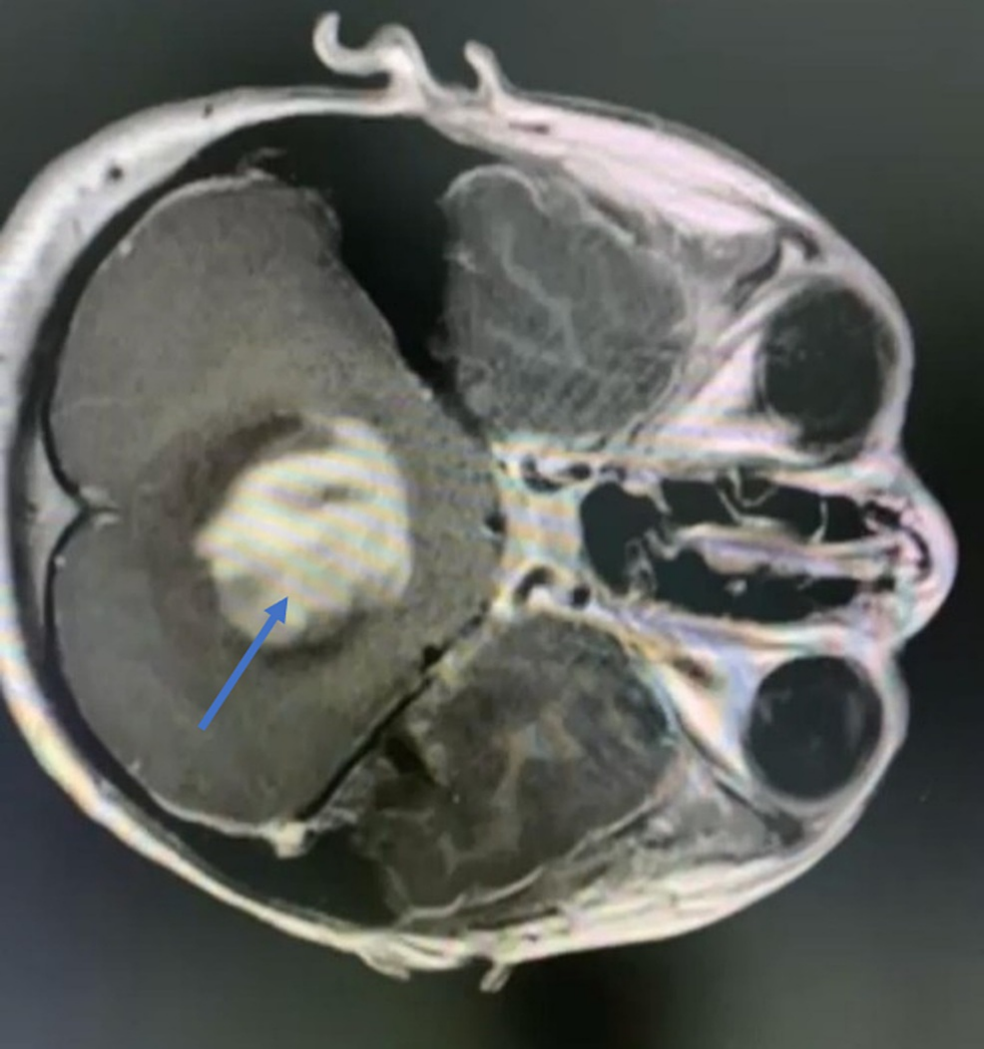
Preoperative image - MRI brain axial cut shows obstructive hydrocephalus

**Figure 3:**
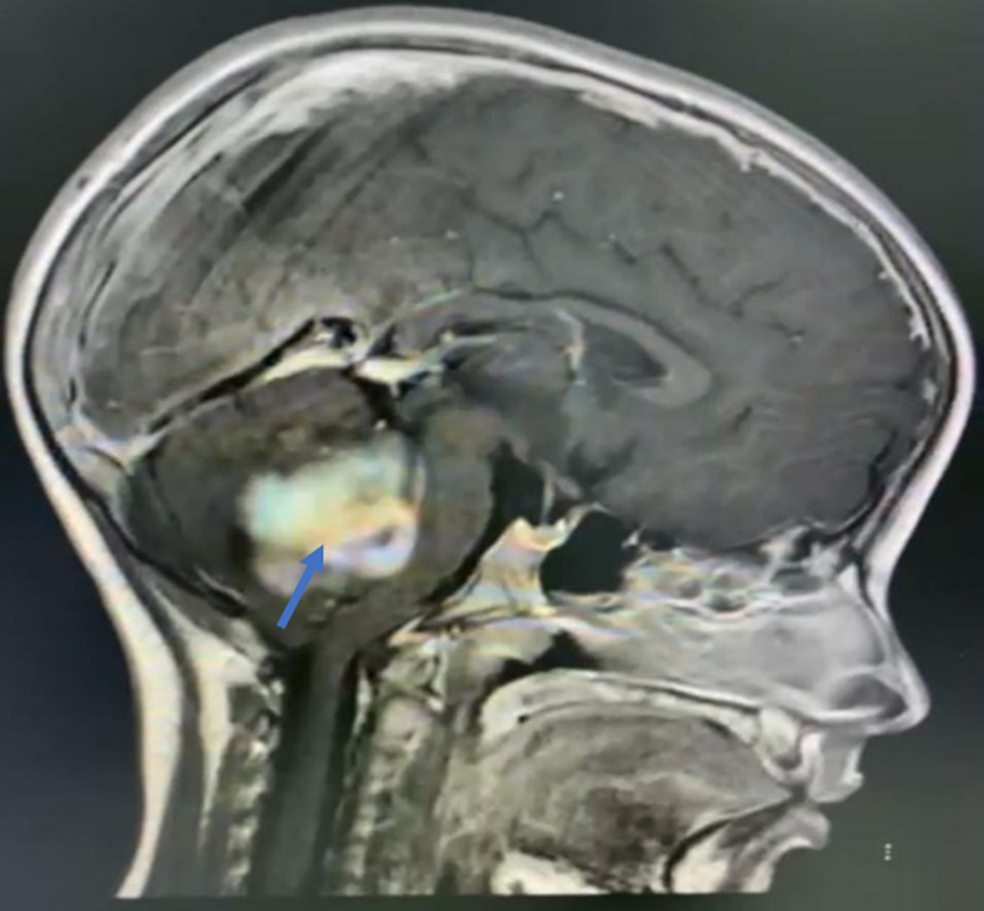
Preoperative image - MRI brain sagittal cut shows obstructive hydrocephalus

**Figure 4:**
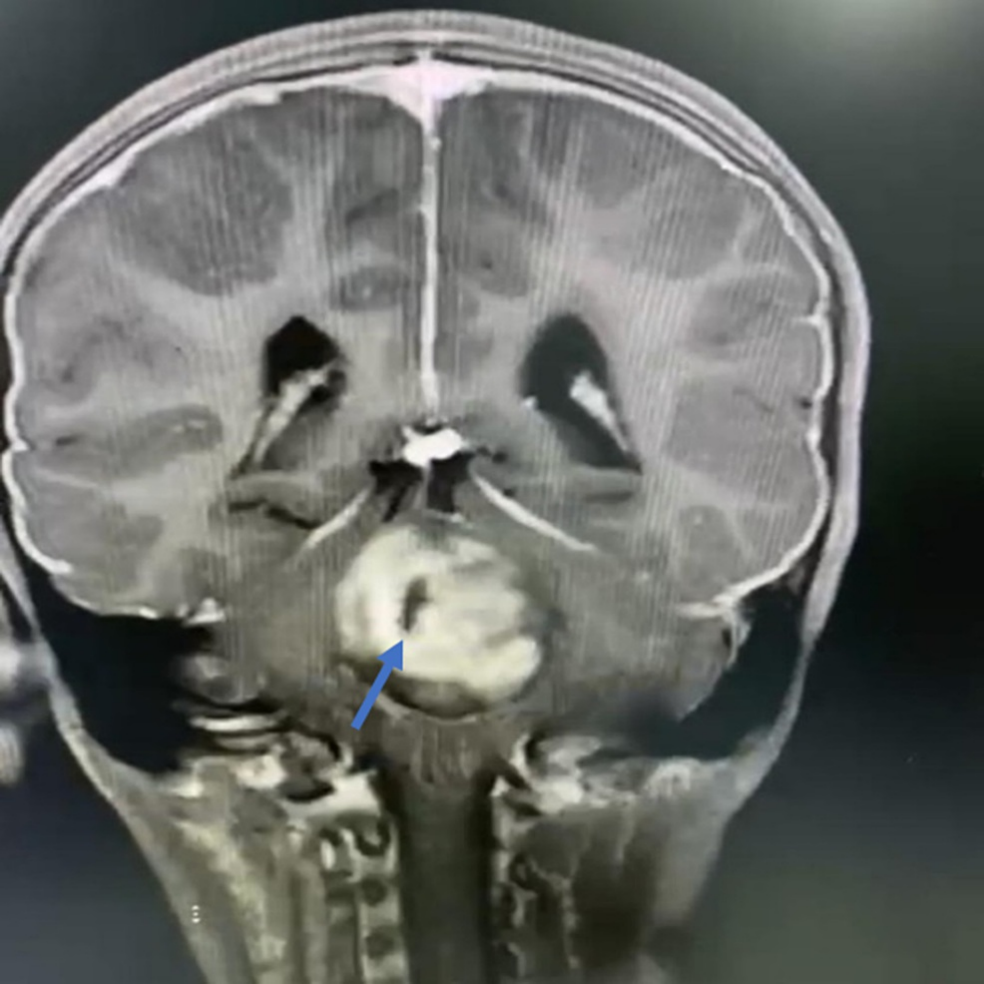
Preoperative image - MRI brain coronal cut shows obstructive hydrocephalus

On initial examination in the ER, the patient’s Glasgow Coma Scale (GCS) score was 14/15; she was slightly drowsy; her pupils were 6 mm bilaterally dilated (she had received dilatation drops at another hospital); she was moving all limbs, with down drifting of the right lower limb; her left gaze showed horizontal diplopia; and she had no nystagmus, no dysmetria, and no dysdiadokokinesia, but she had a positive Romberg sign. A brain MRI scan showed an enhancing mass in the posterior fossa with obstructive hydrocephalus (Figures 2-4).

Timeline and therapeutic intervention

The patient was started on a preoperative workup, a prophylactic antibiotic, dexamethasone (2 mg QID). Consent was obtained, and an emergent right external ventricular drain (EVD) was placed due to the presence of obstructive hydrocephalus and herniation. 

On day one following a right ventriculoperitoneal (VP) shunt insertion, the headache improved. An MRI scan of the whole spine showed a heterogeneous enhancing fourth ventricle/vermian lesion and smooth polymeningeal enhancement, but no metastasis. She was started nil per os (NPO) on fluids, and consent was obtained for a posterior fossa tumor resection. On day two following the right VP insertion (day one following the posterior fossa tumor resection), she was transferred to the pediatric intensive care unit (PICU) for observation, intubated, and her head elevated 30 degrees. Her pupils were 2 mm bilaterally reactive, and she was connected to mechanical ventilation at a mode of pressure cycled assist control (AC/PC) rate of 20, pressure control of 146, and FiO_2_ of 30%. She was administered fentanyl and propofol. Her blood pressure (BP) was 79/50, heart rate (HR) was 110/min, and capillary refill time (CRT) was 2 sec. She was pale, had equal air entry, her abdomen was soft and lax, and she was moving her limbs.

**Figure 5:**
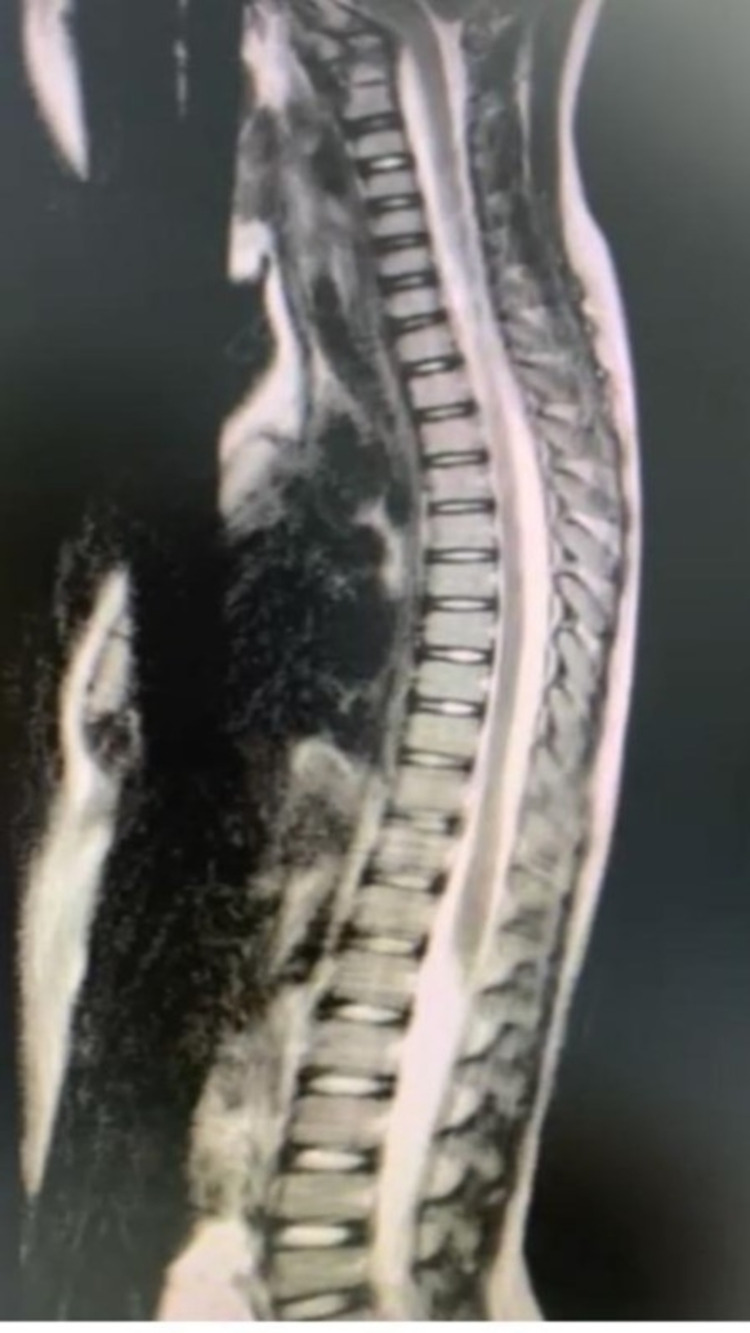
Postoperative image - MRI whole spine

**Figure 6:**
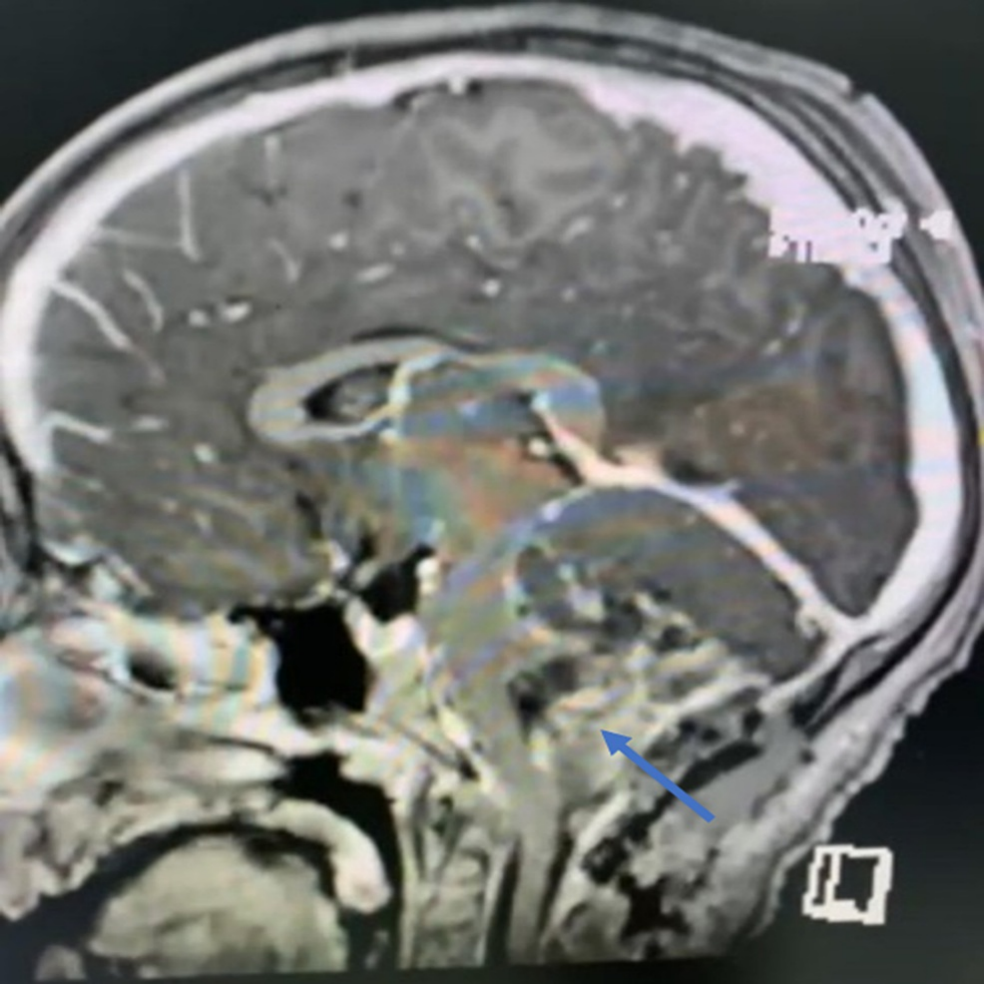
Postoperative image - MRI brain sagittal cut

**Figure 7:**
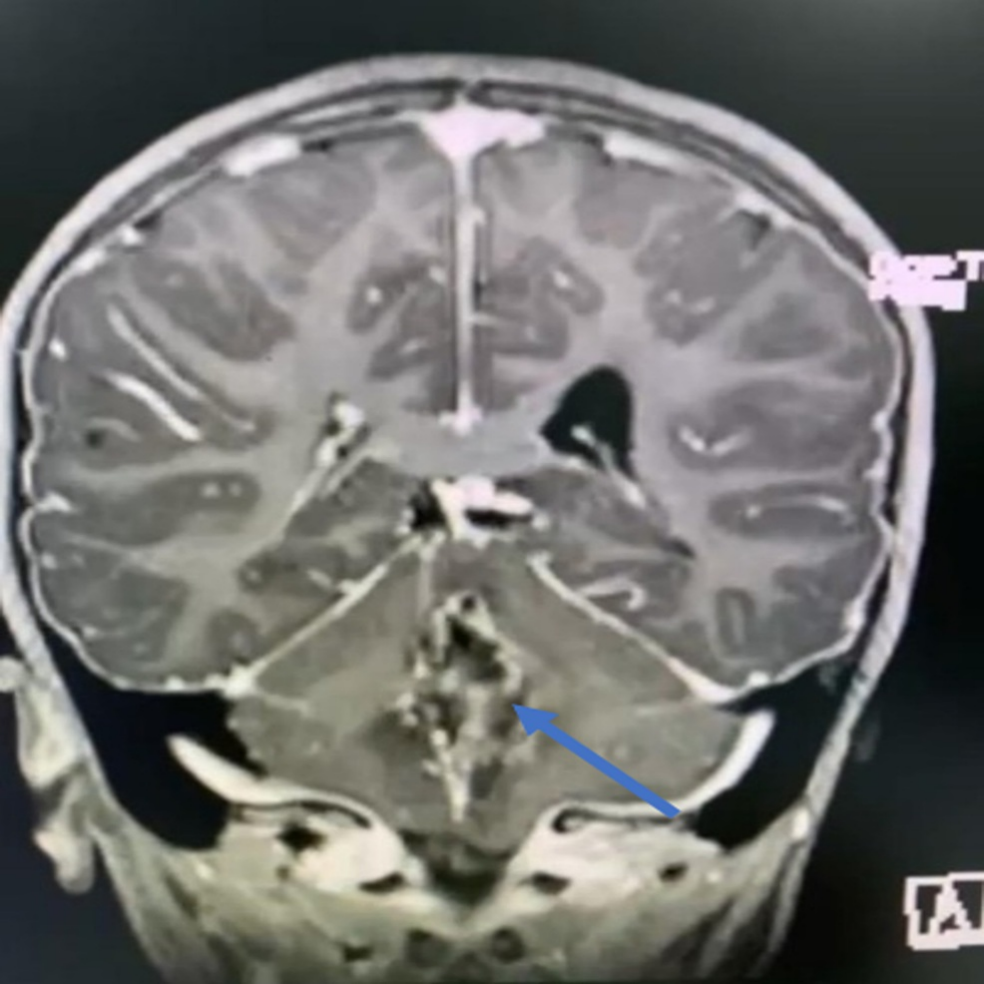
Postoperative image - MRI brain coronal cut

On day two post tumor resection, she was extubated. She was confused and irritable, so a proper neurological examination could not be conducted. Her GCS score was 12/15 (E4, V4, and M4), her pupils were equally reactive, but she showed left upper limb weakness when compared to the right side, and she claimed that she could not see. She had bilateral air entry, coughing, but no crepitation, and a chest x-ray was clear. Her abdomen remained soft and lax. She was continued on IV fluids, fasting, with D5NS fluids plus 10 mEq K, her urine output was positive (UOP) at 2 ml/kg/h, 800 ml, and she vomited four times. She showed no fever spikes and was hemodynamically stable. She was placed on morphine. Her hemoglobin reading was Hb 8.6, so a blood transfusion was done. A brain MRI scan done on the same day, reviewed by two independent neuroradiologists, indicated postoperative changes, with new residual changes around the intracranial catheter and an asymmetric decrease in the size of the right lateral ventricle (Figures 5-7). A diagnosis of cerebellar mutism was established.

On day three post tumor resection, she was less irritable but still aphasic. She was changed to regular pain killers (acetaminophen 318 mg QID, ibuprofen 200 mg BID), and she was discharged to the ward. A physiotherapy referral was made. A nasogastric tube (NGT) was placed as she was drooling, and a nutrition referral was made.

On days five to seven post tumor resection, she was less irritable, had consolable crying, was sleeping well, her GCS score was 12/15, her pupils were 2 mm equal and reactive, but she still showed left upper limb weakness compared to the right. She had episodes of urinary retention, bladder distension, and pain, which had to be relieved multiple times every four to six hours with clean intermittent catheterization (CIC); therefore, a Foley catheter was inserted. The urine culture was negative. A kidney, ureter, and bladder ultrasound (KUB US) pre- and post-voiding showed normal bladder training and urodynamics. A cerebrospinal fluid (CSF) culture was negative. Histopathology revealed a pilocytic astrocytoma grade 1. She was administered dexamethasone (2 mg q8h) for four days with a tapering dose. The urology referral recommendations were CIC q6h.

On day 13 post tumor resection, zolpidem (2.5 mg) was started. On day 15 post tumor resection (day two after zolpidem), she was less irritable, could sit with support, could hold her head unsupported, and had started urinating on her own. A swallowing referral was made because she was drooling and yielded a finding of poor lip sealing and a recommendation to continue with the NGT. Her sutures were removed.

On day 16 post tumor resection (day three after zolpidem), she could turn from side to side on her own and make purposeful movements. On day 17 post tumor resection (day four after zolpidem), she made eye contact. On day 20 post tumor resection (day seven after zolpidem), she could sit unsupported in a wheelchair and could hold her head unsupported. She started to ambulate and walk but remained mute. On day 24 post tumor resection (day 11 after zolpidem), she started to laugh, wave her hands, and shake hands (socially). She was discharged on day 26.

Follow-up and outcomes

On day 51 post tumor resection, the parents were contacted via a virtual clinic because of the coronavirus 2019 (COVID-19) crisis. They reported that the patient was able to sit by herself and could walk with assistance, but she was still mute and unable to swallow.

On day 86 post tumor resection, the parents were contacted again via the virtual clinic because of the COVID-19 crisis. The father reported that the patient had improved 90%. She was able to sit by herself, she could walk independently, and she was now able to swallow and had started to speak.

## Discussion

Cerebellar mutism is a known complication following posterior fossa tumor resection, and it can impact a child’s neurocognition and development [4]. The reasons for the mutism are still being researched but are hypothesized to involve damage to midline structures along the pathway to the cerebrum by too much irrigation or suction [1]. This damage can be reduced by minimal traction of the midline/vermian structures of the cerebellum and by decreasing the amount of tissue resected; however, the risks outweigh the benefits, as the safe resection for decreasing metastasis is less than 1 cm in the posterior fossa [3]. In addition to the technicality of the surgery, some medications have shown efficacy in case reports. Fluvoxamine resulted in improvement of language in patients with no depressive symptoms with unknown mechanism [6]. However, zolpidem and aripiprazole have resulted in improved speech and motor disorders within days of initiation in children with normal learning abilities, which can be explained by stimulation of corticostriatopallidal-thalamocortical pathway as seen also in alleviating negative psychiatric symptoms [7]. However, the present case was challenging. This case had a positive family history of cancers, consanguinity, and hearing problems in siblings, in addition to the child’s learning disability. In the previous studies on normal children, speech was regained in three to four weeks, followed by motor function, which in natural history can stay up to one year [4-6]. In the present study, urinary retention was managed conservatively and fully investigated, but no reason for it was found. However, following treatment with zolpidem, the urinary retention disappeared within one day, unlike reported cases which can improve up to two months with natural history as reported in case series. Another observation was that the patient was fully dependent on an NGT; however, after 2.5-3 months, she was free from the NGT, and there are no reported cases of NGT dependent that underwent successful weaning in the literature [4-6]. She regained motor function by extensive physiotherapy daily, while neurocognition was achieved by increasing quality time with family members through phone calls, messages, video chatting, and in-person visits [8].

## Conclusions

Cerebellar mutism is a known complication of posterior fossa tumor resection and should be mentioned to parents. The management will include a multi-disciplinary approach with extensive involvement of physiotherapists, nutritionists, and ear, nose and throat (ENT) swallow team, as well as administration of some medications like zolpidem. Family support during full recovery is needed.
